# Wind Power Error Estimation in Resource Assessments

**DOI:** 10.1371/journal.pone.0124830

**Published:** 2015-05-22

**Authors:** Osvaldo Rodríguez, Jesús A. del Río, Oscar A. Jaramillo, Manuel Martínez

**Affiliations:** 1 Posgrado en Ingeniería, Universidad Nacional Autónoma de México, Temixco, Morelos, México; 2 Centro de Ciencias de la Complejidad, Universidad Nacional Autónoma de México, Coyoacán, Ciudad de México, México; 3 Instituto de Energías Renovables, Universidad Nacional Autónoma de México, Temixco, Morelos, México; Universidade de Vigo, SPAIN

## Abstract

Estimating the power output is one of the elements that determine the techno-economic feasibility of a renewable project. At present, there is a need to develop reliable methods that achieve this goal, thereby contributing to wind power penetration. In this study, we propose a method for wind power error estimation based on the wind speed measurement error, probability density function, and wind turbine power curves. This method uses the actual wind speed data without prior statistical treatment based on 28 wind turbine power curves, which were fitted by Lagrange's method, to calculate the estimate wind power output and the corresponding error propagation. We found that wind speed percentage errors of 10% were propagated into the power output estimates, thereby yielding an error of 5%. The proposed error propagation complements the traditional power resource assessments. The wind power estimation error also allows us to estimate intervals for the power production leveled cost or the investment time return. The implementation of this method increases the reliability of techno-economic resource assessment studies.

## Introduction

Resource assessment is one of the most important steps in any renewable energy project because it can determine the possible power output, thereby contributing to analyses of the techno-economic feasibility of renewable energy use. Resource assessments are based on weather records and the technical characteristics of the renewable source. This also applies to wind power as a renewable energy source; therefore, the development of accurate and meticulous methods for measuring and monitoring wind speeds are critical factors in its implementation [1].

Typical resource assessment methods involve measuring wind speeds at a frequency of 1 or 2 Hz and recording the arithmetic mean every 10 min for at least one year. These wind speed data are then used to construct a relative frequency distribution, to which a probability density function (*PDF*) is fitted. This function and the specific wind turbine power curve (*WTPC*) are needed to calculate the amount of available energy and the likely electric power output produced in the specific conditions in a region and with the technology employed [2].

The analysis of uncertainty plays an important role in the wind power industry because it can elucidate the error and the degree of reliability in a study, *e.g.*, in the field of wind turbines, the international standard *IEC 61400 part 12-1: Power performance measurements of electricity producing wind turbines* [3] aims “to provide a uniform methodology that will ensure consistency, accuracy and reproducibility in the measurement and analysis of power performance.” Annex D of the standard provides a method for calculating the uncertainty of the wind turbine power performance. During wind turbine power characterization, an extra source of uncertainty is attributable to the statistical process involved in power curve fitting. Given the frequent use of this international standard [3], several studies have aimed to develop improved statistical techniques, such as those used in power performance tests for small wind turbines (*SWT*) and wind power estimation [4–6]. All of these studies recommended techniques for improving the reliability of resource assessments and *SWT* power performance tests.

Statistical calculus is used widely in wind resource assessments; therefore, it is necessary to reduce the sources of uncertainty to obtain reliable assessments [7]. One source of uncertainty is the error associated with the wind speed measurement process, but it is not possible to analyze its effect on wind power production. This information is lost after the arithmetic mean is calculated to construct the mean ensemble, which limits the dispersion concept to the standard deviation for each mean calculated over time. This mean ensemble and the consequent dispersion concept assume that the wind speed with time can be represented by a normal distribution, which is not necessarily true. Several studies have aimed to detect and reduce the sources of uncertainty. Probability distribution models for wind resource variability have been studied [8] and a state of the art method for resource assessment was proposed [9].

To obtain reliable assessments, several techniques are used to measure wind speed, which may complement each other, but no previous studies have considered the quality of the measure employed for power output estimation.

Previously, it was demonstrated that because a longer time is used to obtain the mean ensemble, then the parameters that define the *PDF* may also change [10]. The reliability of the power assessment depends on the accuracy of the *PDF* parameters because they represent the wind speed conditions. However, it is not always easy to obtain accurate estimates due to the limited availability of the data [11], as well as the wide variety of *PDFs* that can be fitted [12, 13].

A previous study considered the influence of the measurement quality on resource assessments [14], but it was assumed that the wind speed mean ensemble follows a normal distribution. In previous research, a wide range of *PDF*s have been used to represent wind speed conditions around the world [12, 13], some of which were represented as bimodal probabilistic models [15].

In terms of the sources of uncertainty related to the goodness-of-fit of the *PDF*s that represent the wind speed conditions, several studies have aimed to determine how well the wind speed data are represented in specific conditions and locations by different PDFs [16–19]. As well as, to obtain reliable resource assessments [10, 12]. However, due to the variable characteristics of the wind speed sampling method, it is impossible to analyze the influence of the error attributable to the meteorological device on the power resource assessment.

Achieving the goal of reliable resource assessments is not an easy task because the process involves several sources of uncertainty, which are related to physical variables and the statistical process used for power estimation. In the present study, we focus on uncertainty analysis during the early stage of power output estimation. Our proposed method may complement the development of reliable resource assessments because it yields an interval for the amount of energy that might be produced. This method involves three variables: wind speed measurements, the goodness-of-fit of the *PDF*, and *WTPC* functions. The output of this method is the error propagation, which considers the *WTPC* and wind speed site conditions represented by a *PDF*, as well as the uncertainty associated with the meteorological device used to measure the wind speeds, where the latter element has never been included in previous methods.

In the first part of this study, to obtain the wind speed error propagation, we present the theoretical framework used to analyze the uncertainties involved in wind power resource assessments. Next, we obtain a general uncertainty expression and we identify all of the elements involved in power estimation, as well as their corresponding uncertainties. These elements provide the theoretical framework of the error propagation method. Finally, several wind speed error propagation percentages are used to assess different wind turbines, thereby calculating different wind power estimates and their corresponding errors.

In this study, we used a wind speed dataset, which was measured and recorded at the Instituto de Energías Renovables U.N.A.M. over a period of 50 days at Temixco, Morelos, México (18º 50’ 23”, 99º 14’ 11”). A Vaisala WXT510 weather transmitter with an accuracy of ±2% installed at 24 m above ground level was used for measurement. The data logger was adjusted to measure and record wind speed at a frequency of 1 Hz. The main characteristic of this wind speed dataset is that it was recorded each second and it had not been subjected to any prior arithmetic treatment. This time period was selected only to illustrate the methodology used to estimate the power error propagation.

## Wind Power Resource Assessment

Wind power resource assessments comprise theoretical processes for estimating the amount of energy that can be generated by considering two specific elements: the wind conditions at the site, which are represented by a *PDF*, and the technical characteristics of the *WTPC* of interest, which are represented by a function *P*_*w*_(*u*).

The corresponding wind energy production for the evaluation period is obtained using Eq (1). This equation is also the most common method employed in *SWT* urban resource assessments [7]. Using this period and a wind regime represented by a *PDF* called *p*, which is a function of the wind *u*, and a known *WTPC* represented by *P*_*w*_(*u*), the average wind machine power, ${\bar{P}}_{w}$, is as follows [2]:

$$\begin{array}{c} {\bar{P}}_{w}=\int_{0}^{\infty }P_{w}\left(u\right)p\left(u\right)du. \end{array}$$ (1)

After this arithmetic process, it is not possible to obtain an error estimate in the resource assessment because all of the dispersion criteria used for the error propagation are mixed. One of these is the inherent dispersion associated with each averaged wind speed and another is the second moment of the *PDF* fitted to the dataset.

In the next section, we explain the procedure used to determine the power output error propagation by considering the wind speed error.

## Error Propagation

In the previous section, we identified the mathematical functions used in power resource assessment. In this section, we present the theoretical framework for error propagation. For a quantity that is a function of at least two measurable variables, *z* = *f*(*x*, *y*, …), the error propagation equation [20, 21] is given by Eq (2).

$$\begin{array}{c} \delta z^{2}≃\delta x^{2}{\left(\frac{\partial z}{\partial x}\right)}^{2}+\delta y^{2}{\left(\frac{\partial z}{\partial y}\right)}^{2}+\ldots +2\delta {xy}^{2}(\frac{\partial z}{\partial x})(\frac{\partial z}{\partial y})+\ldots \end{array}$$ (2)

It is important to mention that expression Eq (2) contains mixed derivatives, which can be eliminated in specific cases when the variables are not correlated. In order to obtain the simplest model of wind error propagation, we assume that this is the case; thus, expression Eq (2) can be rewritten as Eq (3).

$$\begin{array}{c} \delta z^{2}≃\delta x^{2}{\left(\frac{\partial z}{\partial x}\right)}^{2}+\delta y^{2}{\left(\frac{\partial z}{\partial y}\right)}^{2}+\ldots \end{array}$$ (3)

The elements *δx* ^2^ and *δy* ^2^ in expression Eq (3) represent the variance of each variable.

From Eq (1), we define the power density *π*(*u*) as follows:

$$\begin{array}{c} \pi \left(u\right)=P_{w}\left(u\right)p\left(u\right), \end{array}$$ (4)

where *P*_*w*_ represents the wind turbine selected for the power resource assessment and *p*(*u*) is the statistical model that represents the wind speed conditions at the site, as defined previously in the power resource assessment section.

To calculate the power density error, *δπ*, we apply Eq (3) to *π*(*u*). However, the *PDF* that represents the wind speed conditions is a function of three parameters, i.e., the wind speed *u* and two parameters that define the shape *c* and scale *k* of the function. Therefore, the power density *π* is also a function of them, i.e., *π*(*u*, *c*, *k*), as described in Eq (5).

$$\begin{array}{ccc} \delta \pi^{2} & = & {\left(\frac{\partial \pi }{\partial u}\right)}^{2}\delta u^{2}+{\left(\frac{\partial \pi }{\partial c}\right)}^{2}\delta c^{2}+{\left(\frac{\partial \pi }{\partial k}\right)}^{2}\delta k^{2}\\ & = & {\left[\frac{\partial P_{w}}{\partial u}p+P_{w}\frac{\partial p}{\partial u}\right]}^{2}\delta u^{2}+{\left[P_{w}\frac{\partial p}{\partial c}\right]}^{2}\delta c^{2}+{\left[P_{w}\frac{\partial p}{\partial k}\right]}^{2}\delta k^{2} \end{array}$$ (5)

It is important to mention that some of the *PDF*s used in wind resource assessments are only a function of two parameters, i.e., the wind speed *u* and the shape factor *c*. In the present study, we consider only a two-variable *PDF*; therefore, the error propagation expression Eq (5) takes the following form.

$$\begin{array}{ccc} \delta \pi^{2} & = & {\left(\frac{\partial \pi }{\partial u}\right)}^{2}\delta u^{2}+{\left(\frac{\partial \pi }{\partial c}\right)}^{2}\delta c^{2}\\ & = & {\left[\frac{\partial P_{w}}{\partial u}p+P_{w}\frac{\partial p}{\partial u}\right]}^{2}\delta u^{2}+{\left[P_{w}\frac{\partial p}{\partial c}\right]}^{2}\delta c^{2} \end{array}$$ (6)

In Eq (6), there are two known functions, i.e., the WTPC *P*_*w*_ and the wind speed PDF *p*, but two new dispersion elements are included in the wind power error propagation expression: one that corresponds to the wind speed *δu* and a second that corresponds to the parameters used to define the probabilistic model *δc*.

In Eq (6), the wind speed dispersion is represented by *δu*. To calculate this, we need to consider that each wind speed measurement is associated with an inherent uncertainty related to the measurement device *e*_*u*_, which usually has values that range from 1–10%. These uncertainty values correspond to the quality of the measures required by the international standard, which are obtained using first class anemometers and standard meteorological stations. We associate the dispersion concept given by expression Eq (6) with the wind speed uncertainty such that *δu* will contribute important information about the quality of the measure, and thus the quality of the device. The inclusion of this parameter captures the effect of the wind speed error on the power output uncertainty. To the best of our knowledge, this parameter has never been included in previous studies of the resource assessment process. This is possible in our study because the dataset recorded has undergone no previous arithmetic treatment and it preserves the wind speed dispersion information. Thus, *δu* is defined as follows in Eq (7):

$$\begin{array}{c} \delta u_{i}=e_{u}u_{i},\;e_{u}\in \left\{1\%\ldots 10\%\right\}, \end{array}$$ (7)

where *i* is the wind speed recorded at the *i*-th second in the dataset.

We have introduced the elements of Eq (6) and explained *δu*_*i*_. In the next subsection, we identify the specific characteristics of the statistical model that are used to represent the wind speed conditions at the site.

### Rayleigh *PDF*

One of the *PDF*s used most widely in wind resource assessments is the Rayleigh distribution [12], which is the simplest velocity probability distribution because it only requires knowledge of the mean wind speed $\bar{u}$ to calculate the parameter [2]. In this case, the PDF *p*(*u*) and the cumulative distribution function *F*(*u*) are given by:

$$\begin{array}{ccc} p(u) & = & 2cu\exp(-cu^{2}), \end{array}$$ (8)

$$\begin{array}{ccc} F(u) & = & 1-\exp[-cu^{2}], \end{array}$$ (9)

where

$$\begin{array}{ccc} \bar{u} & = & \frac{1}{N}\sum_{i=1}^{N}u_{i}\\ c & = & \frac{\pi }{4{\bar{u}}^{2}}. \end{array}$$ (10)

As mentioned previously, the statistical model used to represent the wind speed conditions at a site may be a function of two or three parameters. To calculate the corresponding error for a two-parameter function, $\bar{u}$ and *c*, as the Rayleigh *PDF*, the final element of the sum in Eq (5) is null because *k* is included in this probabilistic model.

Moreover, the selection of an appropriate statistical model allows us to calculate *δc*, which represents the dispersion of the parameter that determines the shape of the probabilistic function, given that parameter *c* has a well-known associated function, Eq (11). We propagate the uncertainty associated with the speed measurement *e*_*u*_ for the average wind speed defined in Eq (11) using Eq (2), which yields Eq (11).

$$\begin{array}{c} \delta c=e_{u}\frac{1}{n}\sum_{i=1}^{n}u_{i}^{2} \end{array}$$ (11)

Eq (11) contributes extra information about the *PDF* fitted to the general expression for propagation error, which involves a *PDF* criterion, as well as considering the influence of the measurement device in terms of the statistical function used.

The last element required to determine the power resource error propagation is the *WTPC*, which must satisfy the condition of being at least first-order derivable to compute the wind power error propagation. These conditions and their arguments are presented in the next section.

### SWT power curves

In the previous section, we introduced the theoretical elements needed to determine the error propagation in power resource assessments. Next, we present the method used to obtain the final function involved in error propagation, i.e., the *WTPC*, which is represented by *P*_*w*_. This element is known to be a source of uncertainty in resource assessments, so efforts have been made to identify “ideal” power curves [4, 5].

The function *P*_*w*_, which is required to determine the wind power error propagation, must satisfy the following characteristics. The function selected as the power curve *P*_*w*_ must be differentiable, i.e., at least the first derivative must exist because the calculation requires a function that behaves well. It is important to remember that the elements of the *WTPC* must represent it adequately, i.e., the cut in and cut out wind speeds, both of which are equally important because they define the range of operation for the wind turbine that is being analyzed. Therefore, these wind speeds must be well defined in the domain of the *P*_*w*_(*u*) function. In previous studies, it has proved difficult to find continuous and derivable power curves that are appropriate for error propagation analysis, where the usual approach is to find discrete power “curves;” thus, a function must be fitted to a discrete set of points. Finally, the proposed function *P*_*w*_ and the discrete data must be very similar. In order to verify this, we propose that there must be a correlation between the discrete data and the function.

To facilitate the power error calculation, we used a catalog of 28 SWTs and their respective discrete power curves, which are available from the RETScreen project [22]. We refer to these SWTS as small because they all have a nominal power below 5 *kW*.

The *SWP* power curves represented in the RETScreen project are sets of discrete natural values that range between 0 and 30 *m*/*s*. The function *P*_*w*_, which represents each dataset, was calculated by several interpolation functions using different combinations of points. To determine the best fit among the different combinations of points and functions, we calculated Pearson’s correlation coefficient, where the best fit was determined as the coefficient value nearest to one.

In order to satisfy the conditions described above, polynomial extrapolation using Lagrange’s method was employed to fit the discrete wind power data. This method yields a polynomial that satisfies the derivative conditions. It also provides great flexibility when selecting the fixed points, where the polynomial must be defined as the cut in or cut out wind speeds and the maximum power output. Finally, it is possible to test the correlation between the calculated polynomial and the discrete data.

It is possible to use a different interpolation other than that proposed in this study, such as the piecewise cubic spline or piecewise cubic Hermite interpolating polynomial [23], which fits a degree-3 polynomial between two consecutive data. Although the use of Lagrange polynomials introduces error into the power curves, which is analyzed in the supplementary material S2 Table, S1 Fig and S2 Fig, we select the simpler polynomial model for the error propagation calculus.

If there are *N* data values, a polynomial of degree *N* − 1 can be found, which will pass through all the points. This interpolation is known as Lagrange interpolation [24], as described by expression Eq (12):

$$\begin{array}{c} f\left(x\right)=\sum_{i=1}^{N}f\left(x_{i}\right)P_{i}^{L}\left(x\right), \end{array}$$ (12)

where *f*(*x*_*i*_) are the known values of the function and *f*(*x*) is the desired value of the function. The Lagrange polynomial $P_{i}^{L}$ is the polynomial of order *N* − 1, which has a value of 1 when *x* = *x*_*i*_ and 0 for all *x*_*j* ≠ *i*_, as follows.

$$\begin{array}{c} P_{i}^{L}=\frac{\prod_{j\neq i}\left(x-x_{j}\right)}{\prod_{j\neq i}\left(x_{i}-x_{j}\right)} \end{array}$$ (13)

Eq (13) represents the polynomial interpolated by Lagrange’s method, which is the last element needed to calculate the error propagation, i.e., $P_{i}^{L}=P_{w}$. In the next section, we present the results obtained and a discussion.

## Wind power output error in resource estimation

In the previous sections, we presented all the theoretical elements required to determine the error propagation in wind resource assessments. In this section, we present the power curve catalog for the 28 *SWT*s used by *P*_*w*_. Next, we give the results of the power resource assessment and the error propagation related to each assessment. Table 1 presents only the first three polynomials.

**Table 1:** Three power curves fitted by Lagrange’s method. The numbers are assigned in column 1, column 2 shows the name of the *SWT*, and column 3 presents the polynomials fitted using Lagrange’s method.

#	*SWT* Model	Power Curves Fitted by Lagrange’s Method *P*_*w*_(*u*)
1	AWP3.6 (Grid tie) 3.6 m	−1.486 × 10^−6^ *x* ^5^ + 2.594 × 10^−4^ *x* ^4^ − …
	1.6 *kW*	−9.477 × 10^−3^ *x* ^3^ + 1.137 × 10^−1^ *x* ^2^ − …
		… − 3.339 × 10^−1^ *x* + 2.847 × 10^−1^
2	Ampair 600-230 1.7 m	−7.121 × 10^−8^ *x* ^7^ + 4.686 × 10^−6^ *x* ^6^ − …
		… − 1.278 × 10^−4^ *x* ^5^ + 1.908 × 10^−3^ *x* ^4^ − …
		… − 1.726 × 10^−2^ *x* ^3^ + 9.369 × 10^−2^ *x* ^2^ − …
		… − 2.366 × 10^−1^ *x* + 2.097 × 10^−1^
3	Bergey BWCXL1 2.5 m	−5.538 × 10^−7^ *x* ^7^ + 3.533 × 10^−5^ *x* ^6^ − …
	1 *kW*	… − 8.695 × 10^−4^ *x* ^5^ + 1.04 × 10^−2^ *x* ^4^ − …
		… − 6.406 × 10^−2^ *x* ^3^ + 2.108 × 10^−1^ *x* ^2^…
		… − 2.923 × 10^−1^ *x* + 1.359 × 10^−1^

**Fig 1:**
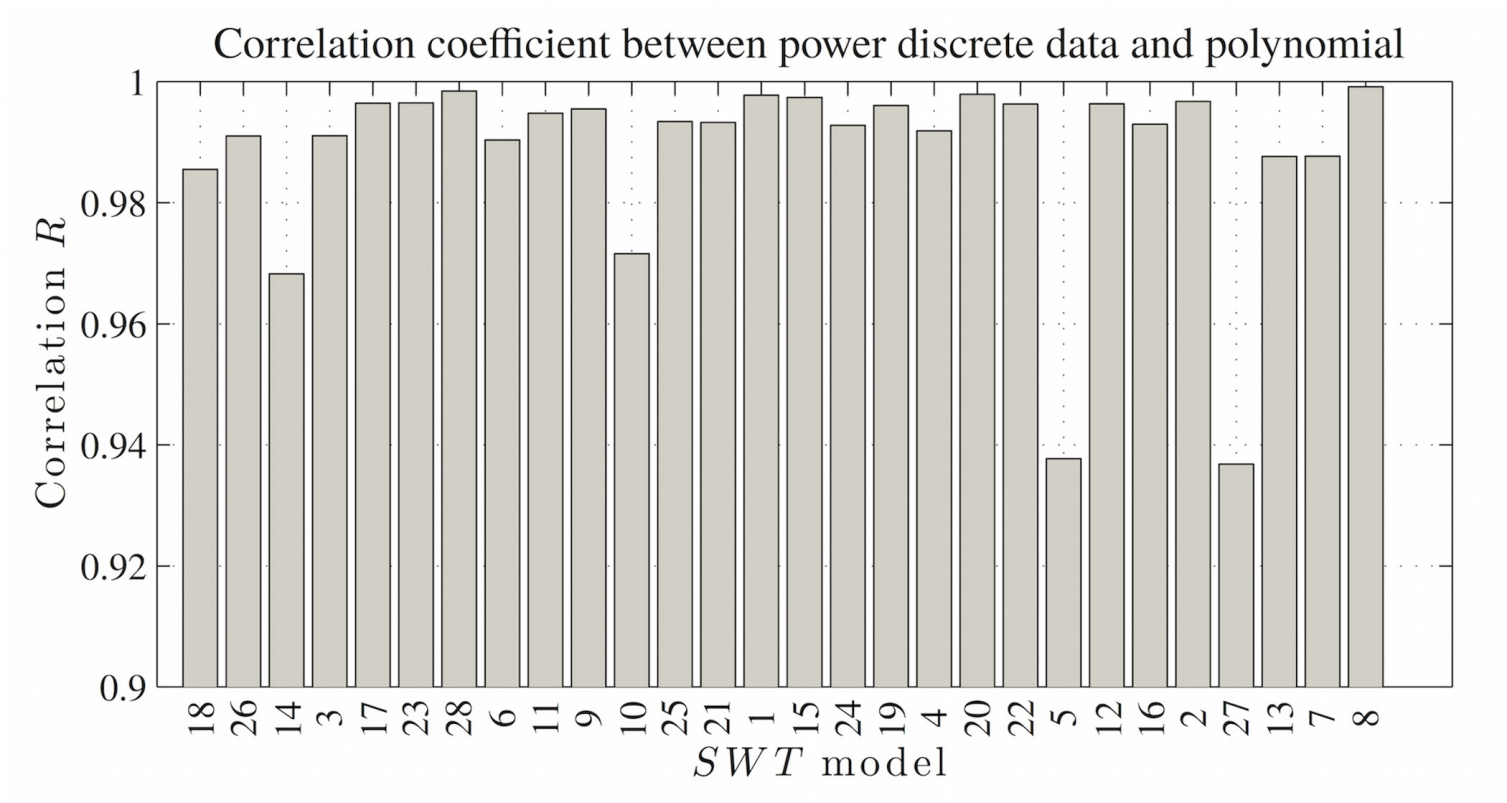
Power curve fitting test. Pearson’s correlation coefficients between the discrete power data and the fitted polynomials. The order of the SWTs correlations is the same as that shown in Fig 3. The different trends in the figures indicate that the calculated power output error was not a simple function of the goodness-of-fit of the polynomials.

In S1 Table Polynomial fitted by Lagrange’s method, comprises the complete catalog of power curves for the wind turbines used in this study, and all of their identification numbers are shown in column 1 of Table 1. The Pearson’s correlation coefficients are plotted for all of the polynomials in Fig 1, which shows that all of the correlation values were greater than 0.93, thereby indicating that the discrete data and fitted polynomials were highly correlated.

**Fig 2:**
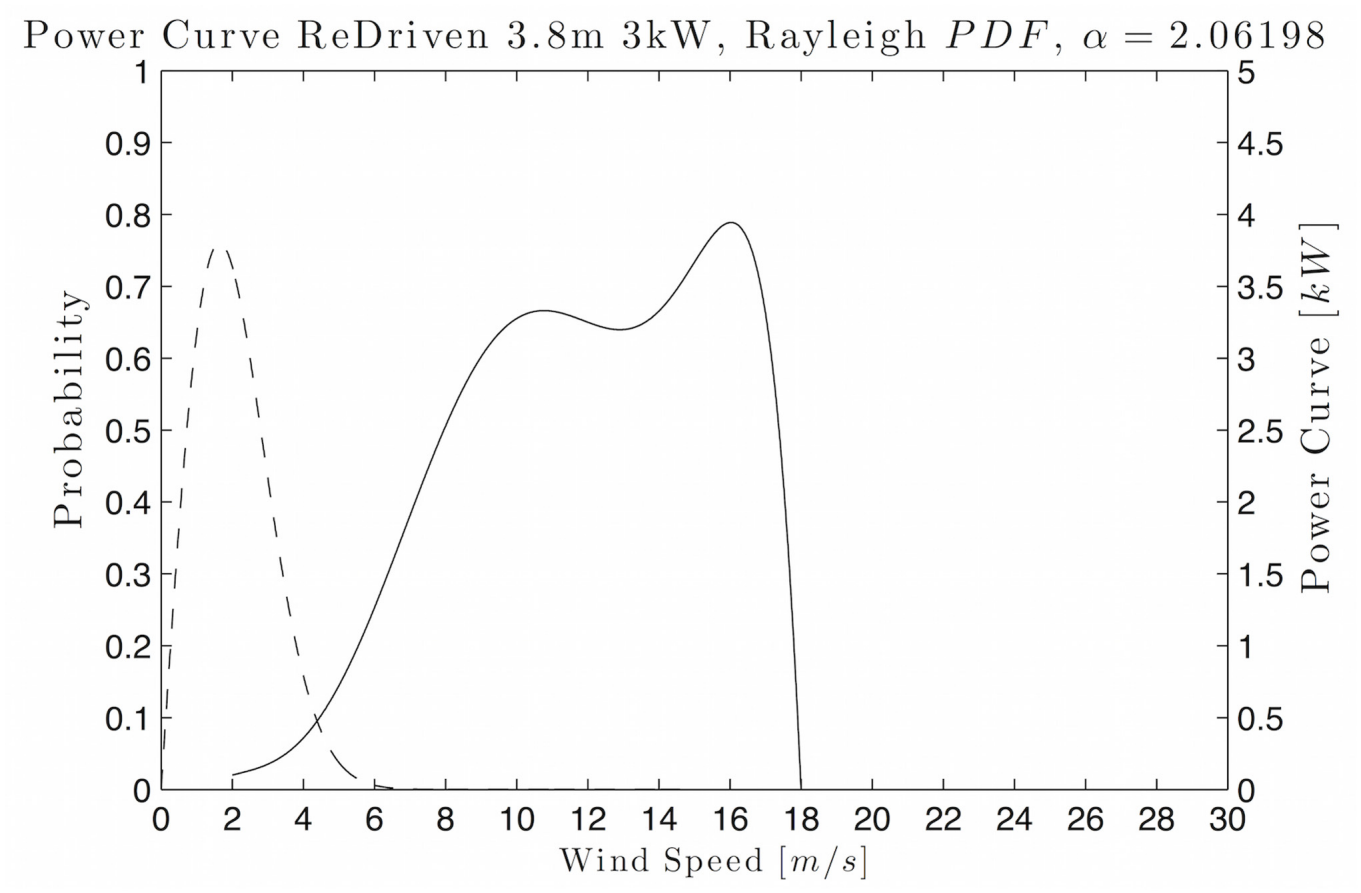
Example of the Rayleigh *PDF* and power curve for resource assessment. The dotted curve represents the Rayleigh *PDF* with parameter *α* = 2.06198. The solid line is the polynomial fitted by Lagrange’s method as the WTPC for the #18 wind turbine model, where this number corresponds to a ReDriven wind turbine with a nominal power of 3 *kW* and a swept area of 3.8 *m*.

For example, Fig 2 shows the polynomial selected as the power curve for the wind turbine *ReDriven 3.8 *m** as a solid line and the Rayleigh function, while the parameter *c* = 2.06198 fitted to the wind data is a dashed line. We can see that the *P*_*w*_ curve is well defined at the cut in and cut out speeds, while it is also a smooth curve, i.e., all of the conditions are satisfied.

**Fig 3:**
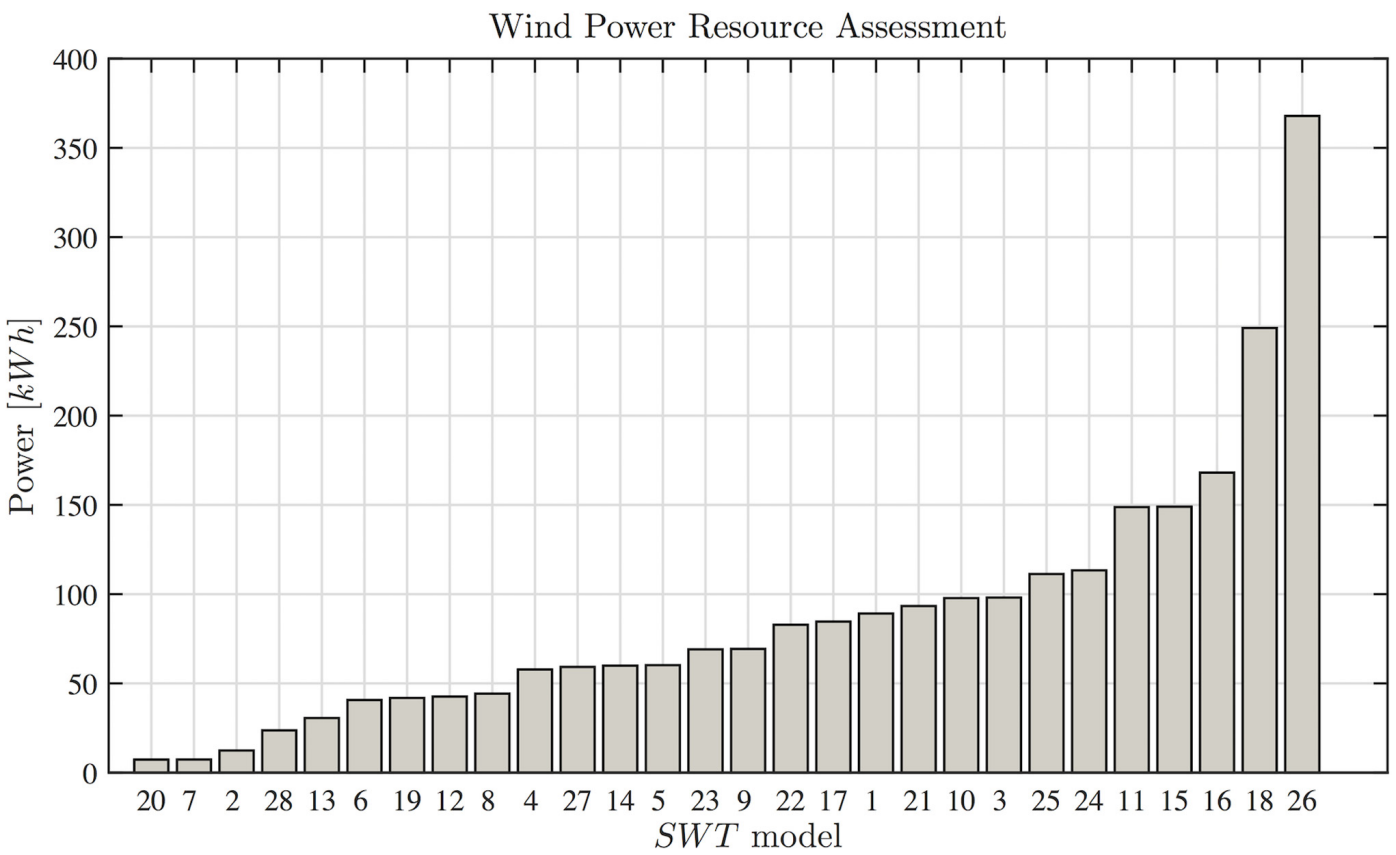
Resource assessments. The power output estimates for the 28 SWTs used in this study. All of the wind turbines are classified as SWTs but the power output results vary greatly. The difference between the highest and lowest outputs is close to 350 *kWh*, and thus selecting an inappropriate device may affect the results of the techno-economic study.

Next, we present the power resource assessments calculated using Eq (1) and the operation time. Fig 3 shows the 28 power resource assessments, which correspond to each of the calculated *SWT* power curves. The power resource assessments are arranged in ascending order.

**Fig 4:**
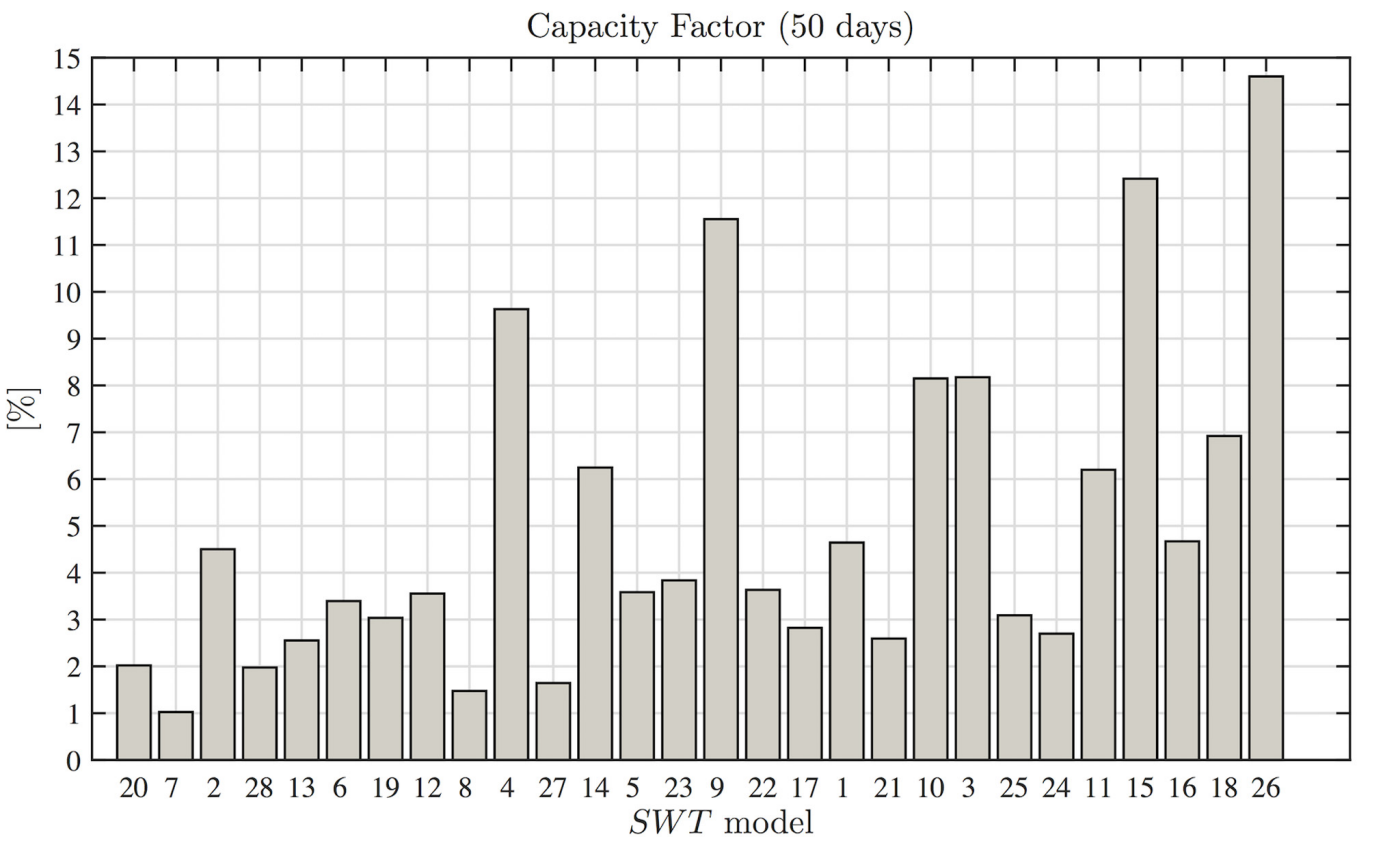
Capacity factor. The capacity factor estimates for the 28 SWTs used in this study complementary to resource assessments. The difference between the highest and lowest capacity factors is close to 13%, and thus selecting an inappropriate device may affect the results of the techno-economic study.

Fig 3 shows that the selection of an appropriate wind turbine is a basic step in resource assessments [6]. All of the wind turbines are for domestic applications, but there is a difference of 300 *kWh* between the lower and higher assessments in exactly the same wind conditions. These assessments correspond to a *Samprey Wren* wind turbine with a diameter of 1 *m* and nominal power of 0.3 *kW*, and a *Travere* wind turbine with nominal power of 2.1 *kW*. Is important to mention, that the turbine with higher power production is not the *SWT* with the higher nominal power in the catalog. To reinforce the idea of the importance of the correct selection of the wind turbine as a key factor in evaluating the resource, the capacity factor is calculated and graphed for each *SWT*, the order in which they appear in Fig 4 is the same as in Fig 3.

**Fig 5:**
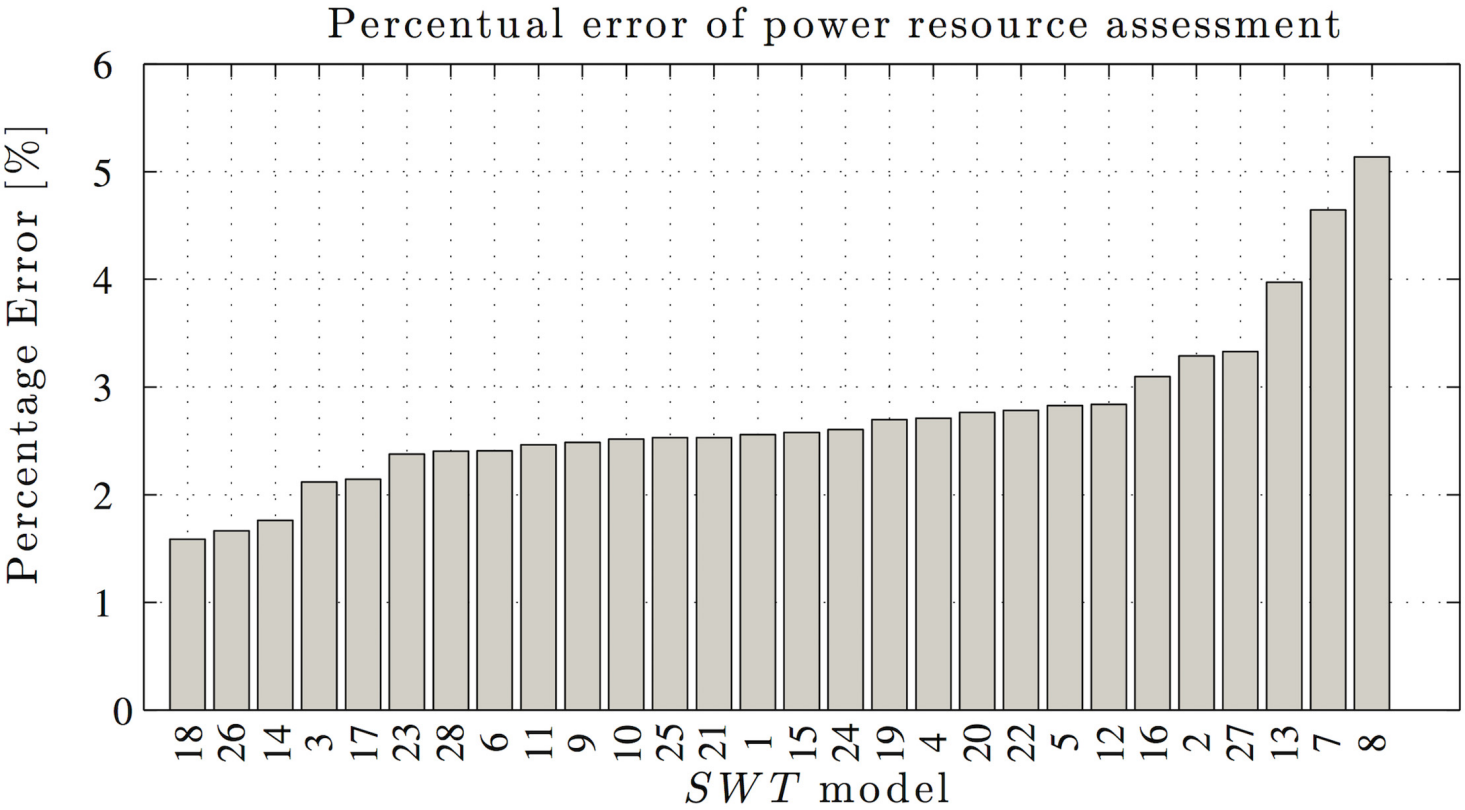
Resource assessment errors. The errors associated with the power output resource assessments calculated for each SWT used in the study.

Finally, we present the propagated errors for the power resource assessments. The results were calculated using Eq (6) and the propagation equation for the sum of all *δπ*(*u*). Fig 5 shows the relative errors calculated for all of the power curves, with a Rayleigh PDF and a device error of 10%. This error is not usual in measurement devices recommended by international standards, but it was included because it represents (in orders of magnitude) errors similar to common weather stations which also provide a maximum point of reference. Wind speed errors of 5% and 2% yielded power output error estimates of 2.5% and 1%, respectively.

As mentioned previously, error propagation comprises the following elements: the power curve, the parameters that define the PDF, and the wind speed dispersion information. However, in error propagation and resource assessments, the unique function that changes is the power curve, and thus it is easy to conclude that the expression used in error propagation is a function of the polynomial. However, Figs 5 and 1 show that the error magnitude considers this fact because there is no observable relationship between the figures, where they do not exhibit similar increases in behavior although they are arranged in the same order of the error associated with the power resource assessments and correlation coefficients.

In a previous study [6], we showed that the mean time had an important effect on wind resource assessments. Thus, the resource assessment was higher if the mean time was low, e.g., the resource assessments differed by about 16% when the mean time was 1 min and 10 min, where we showed that this difference was significant.

The International Standard IEC-61400 [3] provides elements in annex D and E for estimating the uncertainty in measurements, the wind turbine power performance, and the annual energy production. This standard aims to provide a uniform methodology to ensure the consistency, accuracy, and reproducibility of measurements when analyzing the power performance of wind turbines. This procedure involves specific elements in different experimental conditions. However, this calculation has some limitations. First, there is an implicit assumption that: *“the ten minute mean power yield from a wind turbine is fully explained by the simultaneous 10 minute mean wind speed measured at hub height”*, since it has been shown that a 10-min mean wind speed leads to resource underestimation [10]. Second, the error propagation calculation proposed in the present study provides theoretical insights that can only be obtained during the early stage of resource assessment.

### Applications in techno-economic feasibility studies

In the previous section, we described the mathematical basis for error propagation and the error associated with power generation, where these elements contribute to the techno-economic feasibility of electric generation projects. In this sub-section, we demonstrate the usefulness of these concepts and their potential impacts on studies in this area.

A common criterion in the Mexican electric market in terms of the techno-economic feasibility of generation projects, is the calculus of the power production levelized cost *PLC* [25], which is given by Eq (14):

$$\begin{array}{c} PLC=\frac{\sum_{t=-N}^{-1}I_{t}{\left(1+j\right)}^{-t}}{\sum_{t=0}^{n-1}EP{\left(1+j\right)}^{-t}}, \end{array}$$ (14)

where *I*_*t*_ is the investment in the year *t*, *EP* is the annual amount of energy produces, *N* is the construction time, *n* is the economic life of the project, and *j* is the discount rate.

Based on the results presented in the previous section on techno-economic feasibility studies, a useful application of the propagated wind speed error to power estimation is as an extra assessment criterion.

As shown in the previous sections, the main advantage of using a wind speed dataset without statistical treatment is the possibility of estimating the error of *EP*. This energy is calculated by integrating Eq (4) for all of the time measurements, where its associated error *δEP* is calculated by Eq (6) such that *EP* may be expressed as *EP*±*δEP*.

Therefore, the *PLC* calculus can provide an interval of costs, which is given by Eq (15).

$$\begin{array}{c} PLC_{\left\{High,Low\right\}}=\frac{\sum_{t=-N}^{-1}I_{t}{\left(1+j\right)}^{-t}}{\sum_{t=0}^{n-1}EP\pm \delta EP{\left(1+j\right)}^{-t}} \end{array}$$ (15)

This provides an extra element of variability, thereby contributing to the improved reliability of techno-economic studies.

## Conclusions

Resource assessment is a crucial step in any renewable energy project, so anything that contributes to the reduction or detection of the error sources involved in this theoretical process is valuable. In this study, we proposed a general expression and a method to produce a calculus of the wind speed error propagation in terms of power output assessments. We considered three sources of uncertainty involved in the process: wind speed measurements, the fit of the wind speed probability functions, and wind turbine power functions.

Given the characteristics of the discrete wind turbine power curves, a catalog of 28 polynomials was obtained in this study for use in power resource assessments. These power curves behave well and they are defined in terms of the important power curve wind speeds, thereby indicating that the proposed method is suitable.

One of the most important results obtained was the possibility of determining a quantifiable relationship between the wind speed error and the power output error. Using a mean ensemble as the sampling technique for wind resource assessment limits the capacity to analyze the association between the wind dispersion and the mean ensemble, which may be based on an incorrect hypothesis about the normal behavior of the wind speed with time.

All of the wind turbines considered in this study were designed for domestic applications, so selecting an inappropriate device did not have any severe effects on resource assessments in the same wind conditions, but the differences in power production were considerable. Thus, it is important to develop reliable methods for characterizing devices that represent the actual power output behavior.

The effect of the wind speed error propagation on the power output estimate can be calculated using a wind speed dataset without prior statistical treatment, which is useful because it is not possible to interpret this error based on an ensemble of mean wind speeds. Moreover, this approach is useful for understanding the effects of errors on power estimation during the early stage of resource assessments. Furthermore, the results obtained provide an estimate of the measurement quality in order to obtain reliable power estimations because it is possible to establish a limit for the error of the measurement device.

Finally, we presented a useful application in the field of techno-economic feasibility studies based on the results obtained with the propagation method. This application generated an interval for the levelized cost of energy production, which may improve the reliability of the study.

## Supporting Information

S1 Table Polynomial fitted by Lagrange’s method. (PDF)

S2 Table Comparison between cubic spline and Lagrange interpolations. (PDF)

S1 Fig Merit function calculated to determine that Lagrange interpolation is a representative mathematical model. (PDF)

S2 Fig Graphic comparison between Cubic Spline and Lagrange interpolation for the 28 SWTs. (PDF)
