# Supplementary material for: Wind Power Error Estimation in Resource Assessments
Source: PLoS One. 2015 May 22;10(5):e0124830. doi: 10.1371/journal.pone.0124830 (PMC4441467; doi:10.1371/journal.pone.0124830)
Supplement: S1 Table — (PDF) [file pone.0124830.s001.pdf]

S1

Table 2. Polynomial fitted by Lagrange's method

#	*SWT* Model	Power Curves Fitted by Lagrange's Method $p(u)$
1	AWP3.6 (Grid tie) 3.6 m, 1.6 kW	$-1.486\times10^{-6}x^5+2.594\times10^{-4}x^4-9.477\times10^{-3}x^3+0.1137x^2-0.3339x+0.2847$
2	Ampair 600-230 1.7 m	$-7.121\times10^{-8}x^7+4.686\times10^{-6}x^6-1.278\times10^{-4}x^5+1.908\times10^{-3}x^4-\ldots$
		$\ldots-1.726\times10^{-2}x^3+9.369\times10^{-2}x^2-0.2366x+0.2097$
3	Bergey BWCXL1 2.5 m, 1 kW	$-5.538\times10^{-7}x^7+3.533\times10^{-5}x^6-8.695\times10^{-4}x^5+1.04\times10^{-2}x^4-\ldots$
		$\ldots-6.406\times10^{-2}x^3+0.2108x^2-0.2923x+0.1359$
4	Earth-Tech ET500 2.5 m, 500 W	$4.987\times10^{-5}x^4-2.477\times10^{-3}x^3+3.419\times10^{-2}x^2-8.519\times10^{-2}x+5.342\times10^{-2}$
5	Fortis Passaat 3.12 m, 1.4 kW	$-2.112\times10^{-9}x^9+2.372\times10^{-7}x^8-1.138\times10^{-5}x^7+3.048\times10^{-4}x^6-\ldots$
		$\ldots-4.981\times10^{-3}x^5+5.097\times10^{-2}x^4-0.3226x^3+1.206x^2-2.298x+1.679$
6	Future Energy FE1012U 1.8 m, 1 kW	$1.292\times10^{-9}x^9-1.666\times10^{-7}x^8+8.34\times10^{-6}x^7-2.183\times10^{-4}x^6+\ldots$
		$\ldots+3.32\times10^{-3}x^5-3.04\times10^{-2}x^4+0.1663x^3-0.5174x^2+0.8761x-0.62$
7	HEA Energy Ball V100 1.1 m, 0.6 kW	$-5.84\times10^{-10}x^{10}+6.488\times10^{-8}x^9-3.128\times10^{-6}x^8+8.596\times10^{-5}x^7-1.487\times10^{-3}x^6+\ldots$
		$\ldots+1.685\times10^{-2}x^5-0.1262x^4+0.6129x^3-1.831x^2+3.011x-2.035$
8	HEA Energy Ball V200 1.98 m, 2.5 kW	$-4.185\times10^{-9}x^{10}+4.654\times10^{-7}x^9-2.24\times10^{-5}x^8+6.128\times10^{-4}x^7-1.052\times10^{-2}x^6+\ldots$
		$\ldots+0.118x^5-0.8741x^4+4.201x^3-12.46x^2+20.39x-13.75$
9	Hummer 2.7 m, 0.5 kW	$-2.967\times10^{-5}x^4+2.452\times10^{-4}x^3+4.572\times10^{-3}x^2+5.426\times10^{-2}x-0.1283$
10	Hummer 3.1 m, 1 kW	$-8.495\times10^{-7}x^7+5.728\times10^{-5}x^6-1.525\times10^{-3}x^5+2.042\times10^{-2}x^4-0.1472x^3+\ldots$
		$\ldots+0.581x^2-1.044x+0.6613$
11	Hummer 3.8 m, 2 kW	$-2.223\times10^{-9}x^9+1.976\times10^{-7}x^8-6.513\times10^{-6}x^7+8.174\times10^{-5}x^6+3.685\times10^{-4}x^5-\ldots$
		$\ldots-2.228\times10^{-2}x^4+0.251x^3-1.224x^2+2.862x-2.493$
12	Joliet Cyclone 1 2.7 m, 1 kW	$-1.746\times10^{-5}x^4-4.73\times10^{-4}x^3+1.909\times10^{-2}x^2-5.479\times10^{-2}x+3.619\times10^{-2}$
13	Joliet Ultra X900 3 m, 1 kW	$-9.524\times10^{-6}x^4-8.222\times10^{-4}x^3+2.39\times10^{-2}x^2-7.544\times10^{-2}x+5.238\times10^{-2}$
14	Kestrel e230 2.3 m, 0.8 kW	$-1.406\times10^{-7}x^7+7.58\times10^{-6}x^6-1.435\times10^{-4}x^5+1.034\times10^{-3}x^4-1.278\times10^{-3}x^3-\ldots$
		$\ldots-4.66\times10^{-3}x^2+3.14\times10^{-2}x-2.636\times10^{-2}$
15	Kestrel 300i 3 m, 1 kW	$-6.97\times10^{-12}x^{12}+1.076\times10^{-9}x^{11}-7.351\times10^{-8}x^{10}+2.93\times10^{-6}x^9-7.549\times10^{-5}x^8+\ldots$
		$\ldots+1.318\times10^{-3}x^7-1.587\times10^{-2}x^6+0.1321x^5-0.7472x^4+2.77x^3-6.254x^2+7.51x-3.396$
16	Kestrel 400i 4 m, 3 kW	$-1.289\times10^{-8}x^9+1.509\times10^{-6}x^8-7.491\times10^{-5}x^7+2.053\times10^{-3}x^6-3.387\times10^{-2}x^5+\ldots$
		$\ldots+0.3441x^4-2.125x^3+7.6x^2-13.96x+9.966$

#	SWT Model	Power Curves Fitted by Lagrange's Method $p(u)$
17	Kingspan KW3 3.8 m, 2.5 kW	$-1.317 \times 10^{-10}x^{10} + 1.967 \times 10^{-8}x^9 - 1.264 \times 10^{-6}x^8 + 4.575 \times 10^{-5}x^7 - \ldots$
		$\ldots - 1.027 \times 10^{-3}x^6 + 1.481 \times 10^{-2}x^5 - 0.1375x^4 + 0.7969x^3 - 2.669x^2 + 4.692x - 3.297$
18	ReDriven 3.8 m, 3 kW	$-2.214 \times 10^{-6}x^7 + 1.094 \times 10^{-4}x^6 - 1.93 \times 10^{-3}x^5 + 1.369 \times 10^{-2}x^4 - \ldots$
		$\ldots - 2.432 \times 10^{-2}x^3 - 6.558 \times 10^{-2}x^2 + 0.3148x - 0.2368$
19	Samprey Mistral 2 m, 1.15 kW	$-2.411 \times 10^{-9}x^9 + 2.707 \times 10^{-7}x^8 - 1.288 \times 10^{-5}x^7 + 3.384 \times 10^{-4}x^6 - 5.354 \times 10^{-3}x^5 + \ldots$
		$\ldots + 5.224 \times 10^{-2}x^4 - 0.3102x^3 + 1.073x^2 - 1.897x + 1.299$
20	Samprey Wren 1 m, 0.3 kW	$-4.992 \times 10^{-10}x^9 + 5.403 \times 10^{-8}x^8 - 2.466 \times 10^{-6}x^7 + 6.183 \times 10^{-5}x^6 - 9.306 \times 10^{-4}x^5 + \ldots$
		$\ldots + 8.642 \times 10^{-3}x^4 - 4.926 \times 10^{-2}x^3 + 0.1677x^2 - 0.2995x + 0.2099$
21	Samrey Merlin 3.5 m, 3 kW	$-5.529 \times 10^{-9}x^9 + 6.113 \times 10^{-7}x^8 - 2.852 \times 10^{-5}x^7 + 7.293 \times 10^{-4}x^6 - 1.112 \times 10^{-2}x^5 + \ldots$
		$\ldots + 0.1031x^4 - 0.5732x^3 + 1.842x^2 - 3.009x + 1.896$
22	Skystream 3.7 m, 1.9 kW	$-8.027 \times 10^{-10}x^9 + 4.651 \times 10^{-8}x^8 + 2.47 \times 10^{-7}x^7 - 8.491 \times 10^{-5}x^6 + 2.876 \times 10^{-3}x^5 - \ldots$
		$\ldots - 4.647 \times 10^{-2}x^4 + 0.4023x^3 - 1.808x^2 + 3.987x - 3.302$
23	Sonkyo Windspot 4.05 m, 1.5 kW	$4.828 \times 10^{-9}x^9 - 5.211 \times 10^{-7}x^8 + 2.313 \times 10^{-5}x^7 - 5.564 \times 10^{-4}x^6 + 8.011 \times 10^{-3}x^5 - \ldots$
		$\ldots - 0.07178x^4 + 0.3988x^3 - 1.298x^2 + 2.29x - 1.655$
24	Sonkyo Windspot 4.05 m, 3.5 kW	$-1.756 \times 10^{-6}x^7 + 1.152 \times 10^{-4}x^6 - 2.987 \times 10^{-3}x^5 + 3.904 \times 10^{-2}x^4 - \ldots$
		$\ldots - 0.2769x^3 + 1.107x^2 - 2.167x + 1.585$
25	Travere 3.6 m, 3 kW	$9.291 \times 10^{-11}x^{10} - 1.445 \times 10^{-8}x^9 + 9.521 \times 10^{-7}x^8 - 3.462 \times 10^{-5}x^7 + 7.579 \times 10^{-4}x^6 -$
		$\ldots\ldots - 1.022 \times 10^{-2}x^5 + 8.345 \times 10^{-2}x^4 - 0.3939x^3 + 1.019x^2 - 1.179x + 0.3795$
26	Travere 6 m, 2.1 kW	$2.548 \times 10^{-12}x^{11} - 5.55 \times 10^{-10}x^{10} + 5.045 \times 10^{-8}x^9 - 2.549 \times 10^{-6}x^8 + 7.962 \times 10^{-5}x^7 - \ldots$
		$\ldots - 1.605 \times 10^{-3}x^6 + 2.104 \times 10^{-2}x^5 - 0.1756x^4 + 0.8809x^3 - 2.392x^2 + 3.222x - 1.555$
27	Westwind 3.7 m, 3 kW	$-3.94 \times 10^{-8}x^9 + 3.585 \times 10^{-6}x^8 - 1.393 \times 10^{-4}x^7 + 3.019 \times 10^{-3}x^6 - 0.04x^5 + 0.3332x^4 - \ldots$
		$\ldots - 1.729x^3 + 5.369x^2 - 8.936x + 6.0$
28	Zephyr Airdolphin 1.8 m, 1 kW	$1.425 \times 10^{-10}x^9 - 2.931 \times 10^{-8}x^8 + 1.847 \times 10^{-6}x^7 - 5.477 \times 10^{-5}x^6 + 8.77 \times 10^{-4}x^5 - \ldots$
		$\ldots - 8.035 \times 10^{-3}x^4 + 4.305 \times 10^{-2}x^3 - 0.1238x^2 + 0.1922x - 0.1297$
