# Supplementary material for: Wind Power Error Estimation in Resource Assessments
Source: PLoS One. 2015 May 22;10(5):e0124830. doi: 10.1371/journal.pone.0124830 (PMC4441467; doi:10.1371/journal.pone.0124830)
Supplement: S2 Table — (PDF) [file pone.0124830.s002.pdf]

S2

SWT number	SWT Model	Number of coefficients Lagrange	Number of coefficients Cubic Spline
1	AWP3.6(Grid tie) 3.6m 1.6kw	5	76
2	Ampair 600-230 1.7m	7	72
3	Bergey BWCXL1 2.5m 1kw	7	80
4	Earth-Tech ET500 2.5m 500w	4	92
5	Fortis Passaat 3.12m 1,4kW	9	96
6	Future Energy FE1012U 1.8m 1kW	9	76
7	HEA Energy Ball V100 1.1m 0.6kW	10	80
8	HEA Energy Ball V200 1.98m 2.5kW	10	20
9	Hummer 2.7m 0.5kw	4	76
10	Hummer 3.1m 1kw	7	76
11	Hummer 3.8m 2kW	9	96
12	Joliet Cyclone 1 2.7m 1kw	4	80
13	Joliet Ultra X900 3m 1kw	4	80
14	Kestrel e230 2.3m 0.8kw	7	80
15	Kestrel 300i 3m 1kW	12	96
16	Kestrel 400i 4m 3kW	9	96
17	Kingspan KW3 3.8m 2.5kW	10	116
18	ReDriven 3.8m 3kw	7	72
19	Samprey Mistral 2m 1.15kw	9	96
20	Samprey Wren 1m 0.3kw	9	92
21	Samrey Merlin 3.5m 3kw	9	96
22	Skystream 3.7m 1.9kw	9	96
23	Sonkyo Windspot 1.5kW 4.05m	9	76
24	Sonkyo Windspot 3.5kW 4.05m	7	76
25	Travere 3.6m 3kW	9	112
26	Travere 6m 2.1kW	11	120
27	Westwind 3.7m 3kw	9	76
28	Zephyr Airdolphin 1.8m 1kW	9	96

Table 3. Lagrange interpolation was proposed to represent wind turbine power curves since this method fits a function not only with the characteristics needed in error propagation, but also a mathematical model with fewer coefficients compared to Cubic Spline interpolation. Here, we present the number of coefficients to interpolate each of the wind turbines of the catalog using both interpolations.
