# Supplementary material for: Wind Power Error Estimation in Resource Assessments
Source: PLoS One. 2015 May 22;10(5):e0124830. doi: 10.1371/journal.pone.0124830 (PMC4441467; doi:10.1371/journal.pone.0124830)
Supplement: S1 Fig — (PDF) [file pone.0124830.s003.pdf]

S3

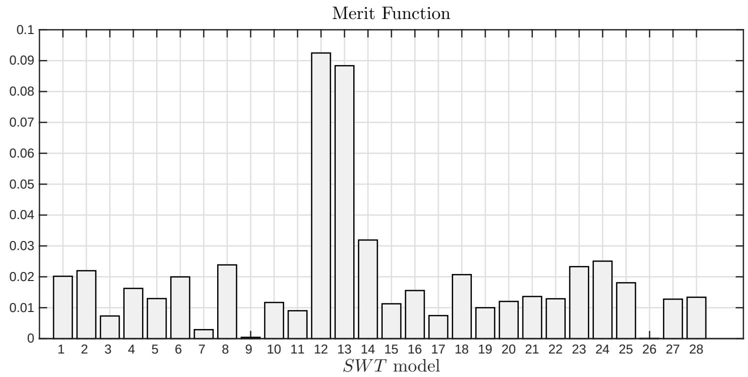

Figure 6. Merit function values of the 28 *SWT* catalog. Merit function is calculated to determine if the Lagrange interpolation is a representative mathematical model of the RETScreen data sets. All values are near zero, therefore are valid mathematical representations of the data sets, with exception of #12 and #13. Therefore, although a Cubic Spline interpolation will result in a better representation of the power curve, Lagrange interpolation offers a simpler mathematical model to calculate error propagation.
