# Supplementary material for: Wind Power Error Estimation in Resource Assessments
Source: PLoS One. 2015 May 22;10(5):e0124830. doi: 10.1371/journal.pone.0124830 (PMC4441467; doi:10.1371/journal.pone.0124830)
Supplement: S2 Fig — (PDF) [file pone.0124830.s004.pdf]

S4

Graphic comparison between Cubic Spline and Lagrange interpolation for the 28 SWT

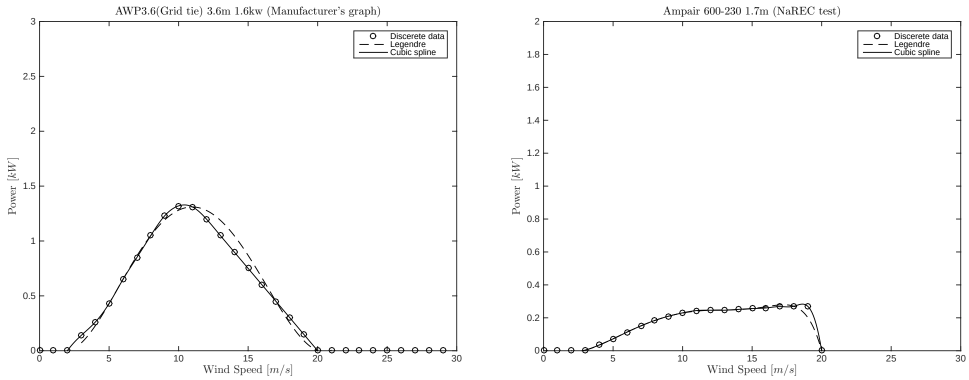

(a) AWP3.6 **(b)** Ampair

Figure 7. Comparison between Cubic Spline and Lagrange interpolation, graphed in solid and dashed line respectively.

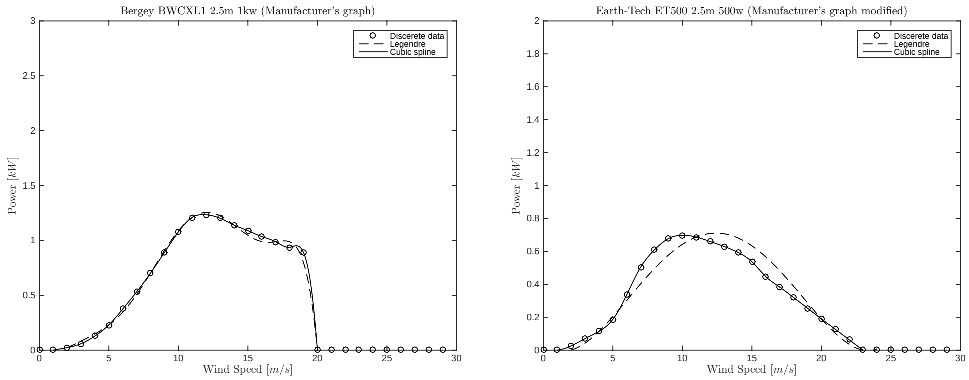

(a) Bergey **(b)** Earth-Tech

Figure 8. Comparison between Cubic Spline and Lagrange interpolation, graphed in solid and dashed line respectively.

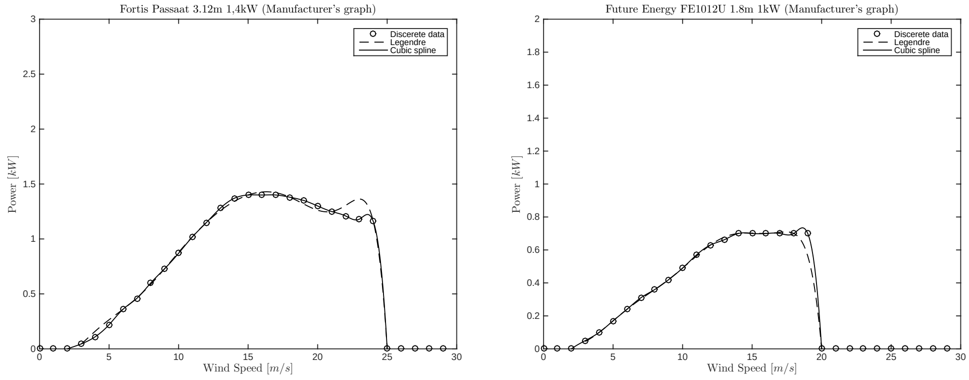

(a) Fortis Passaat **(b)** Future Energy

Figure 9. Comparison between Cubic Spline and Lagrange interpolation, graphed in solid and dashed line respectively.

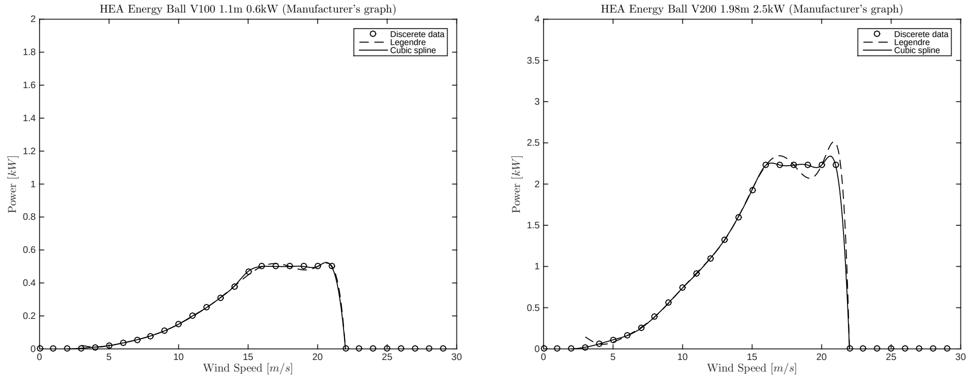

(a) HEA Energy Ball V100 **(b)** HEA Energy Ball V200

Figure 10. Comparison between Cubic Spline and Lagrange interpolation, graphed in solid and dashed line respectively.

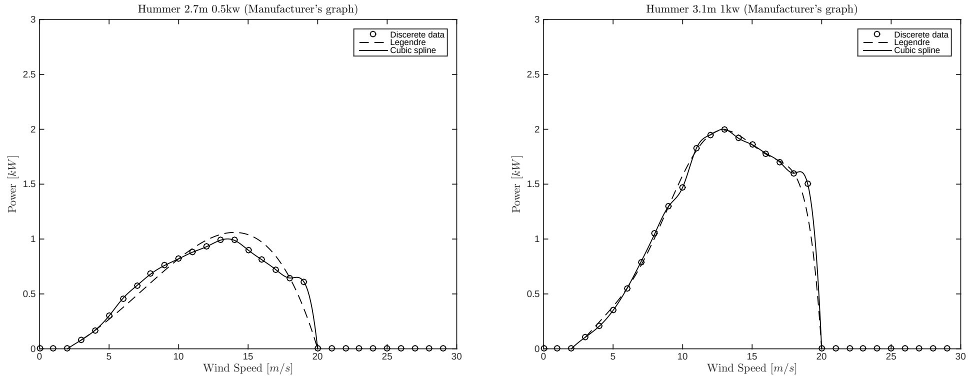

(a) Hummer 2.7 **(b)** Hummer 3.1

Figure 11. Comparison between Cubic Spline and Lagrange interpolation, graphed in solid and dashed line respectively.

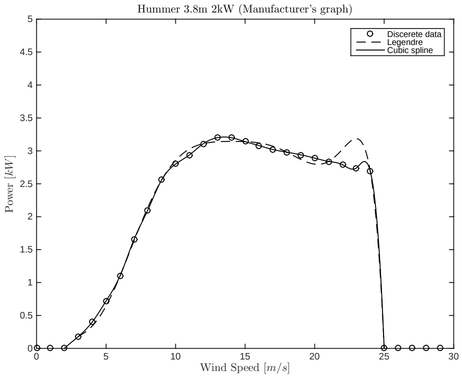

(a) Hummer 3.8

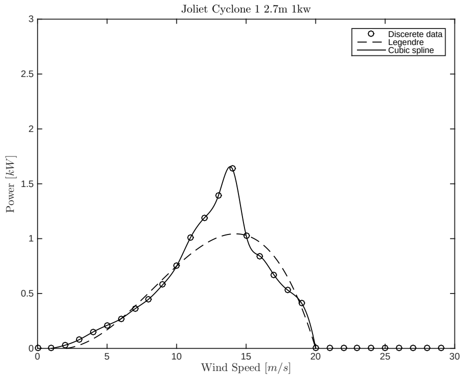

(b) Joliet Cyclone

Figure 12. Comparison between Cubic Spline and Lagrange interpolation, graphed in solid and dashed line respectively.

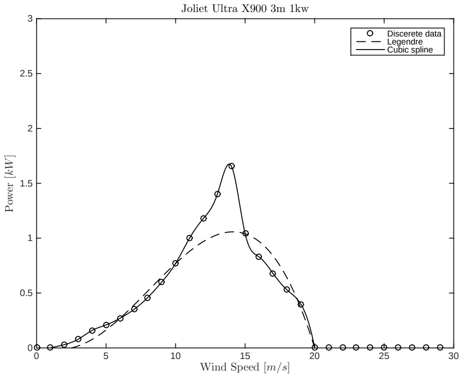

(a) Joliet Ultra

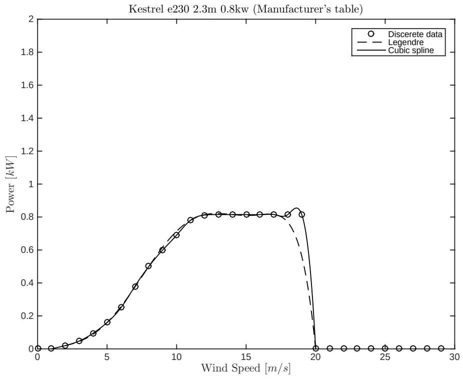

(b) Kestrel e 230

Figure 13. Comparison between Cubic Spline and Lagrange interpolation, graphed in solid and dashed line respectively.

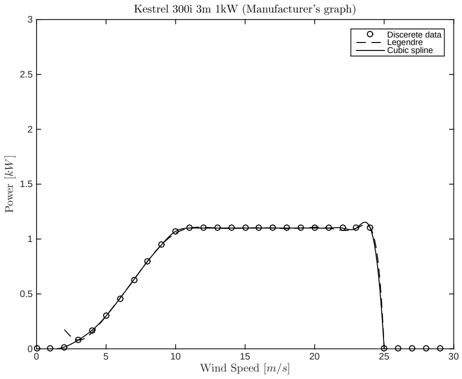

(a) Kestrel 300i

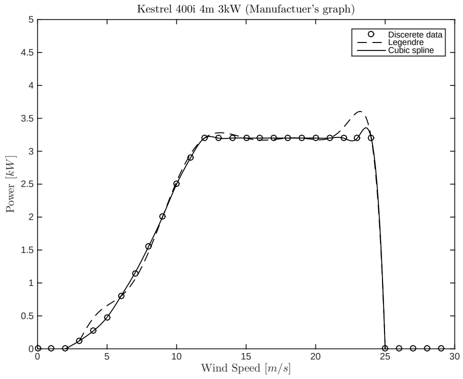

(b) Kestrel 400i

Figure 14. Comparison between Cubic Spline and Lagrange interpolation, graphed in solid and dashed line respectively.

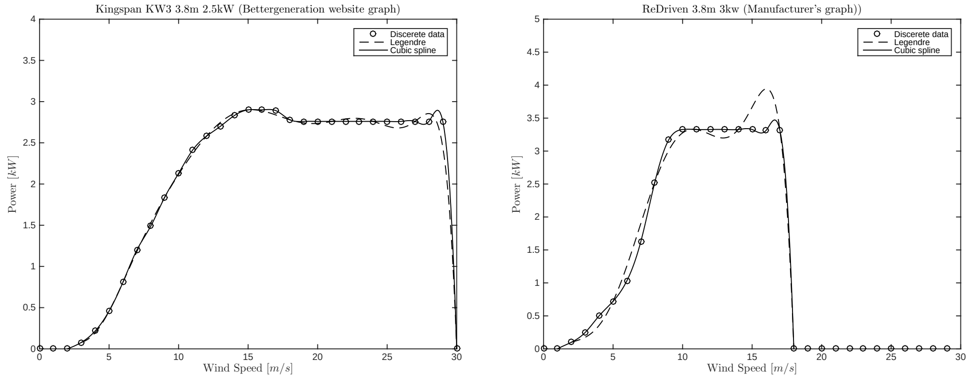

(a) Kingspan **(b)** ReDriven

Figure 15. Comparison between Cubic Spline and Lagrange interpolation, graphed in solid and dashed line respectively.

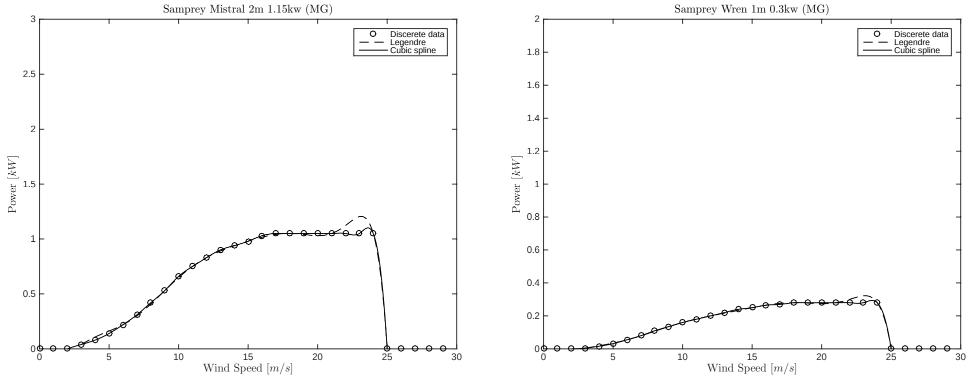

(a) Samprey Mistral **(b)** Samprey Wren

Figure 16. Comparison between Cubic Spline and Lagrange interpolation, graphed in solid and dashed line respectively.

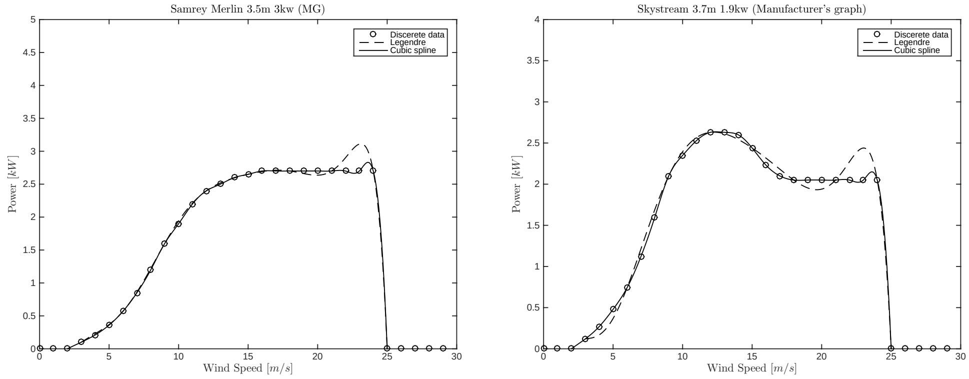

(a) Samprey Merlin **(b)** Skysteram

Figure 17. Comparison between Cubic Spline and Lagrange interpolation, graphed in solid and dashed line respectively.

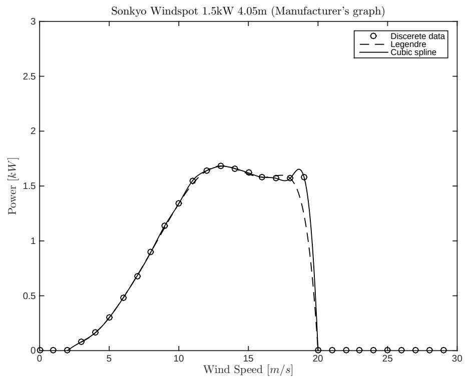

(a) Sonkyo Windspot 1.5

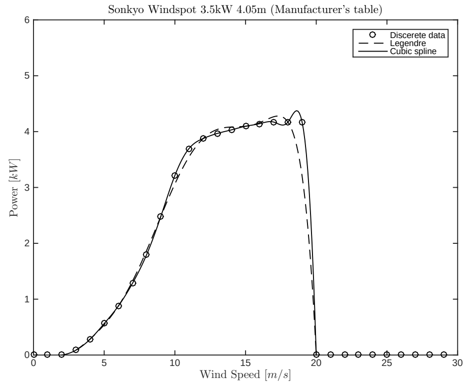

(b) Sonkyo Windspot 3.5

Figure 18. Comparison between Cubic Spline and Lagrange interpolation, graphed in solid and dashed line respectively.

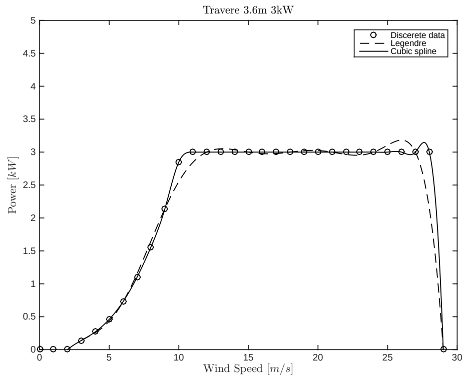

(a) Travere 3.6

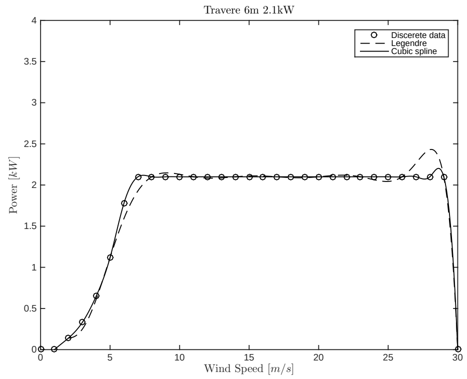

(b) Travere 6

Figure 19. Comparison between Cubic Spline and Lagrange interpolation, graphed in solid and dashed line respectively.

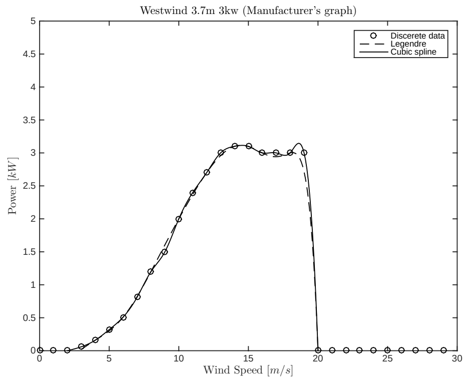

(a) Westwind

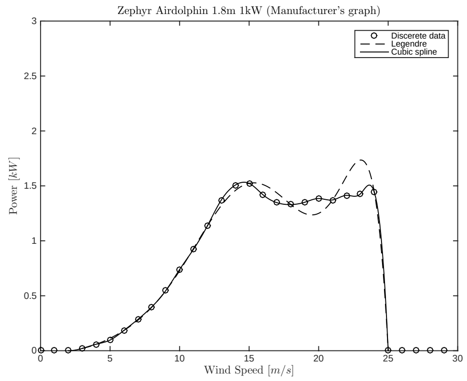

(b) Zephyr Airdolphin

Figure 20. Comparison between Cubic Spline and Lagrange interpolation, graphed in solid and dashed line respectively.
